# Attenuation of canine distemper virus leads to a potent antiviral innate immune response with restricted infection of alveolar macrophages

**DOI:** 10.1128/jvi.01761-25

**Published:** 2025-12-17

**Authors:** Pauline Pöpperl, Elisa Chludzinski, Melanie Stoff, Robert Geffers, Martin Ludlow, Andreas Beineke

**Affiliations:** 1 Department of Pathology, University of Veterinary Medicine Hannover, Foundation 26556 , Hanover, Germany; 2 Center for Systems Neuroscience (ZSN), Hanover, Germany; 3 Genome Analytics, Helmholtz Centre for Infection Research 28336, Brunswick, Germany; 4 Research Center for Emerging Infections and Zoonoses, University of Veterinary Medicine Hannover, Foundation 26556 , Hanover, Germany; University Medical Center Freiburg, Freiburg, Germany

**Keywords:** canine distemper virus, alveolar macrophages, vaccines, innate immunity

## Abstract

Canine distemper virus (CDV, species *Morbillivirus canis*) is a highly contagious pathogen with a broad host range among carnivores. In common with measles virus, alveolar macrophages (AMs) are among the first target cells of infection in the respiratory tract. Therefore, *in vitro* infections of primary canine AMs were performed with the attenuated Onderstepoort (Ond) and field R252 strain of CDV over a period of 6 days. This showed that AMs are permissive to CDV infection and that such infections are productive with respect to the release of new virus particles. Phenotypic differences were observed over the entire course of the experiment, as higher levels of infection and virus production were observed in CDV R252-infected AMs, while infection with CDV Ond resulted in more prominent cytopathic effects, including syncytium formation. Transcriptome analyses of samples from 1 day post-infection via total RNA sequencing demonstrated further marked differences with respect to the pro-inflammatory response and cell death pathways. CDV Ond-infected AMs exhibited robust induction of pro-inflammatory mediators including type I interferon-related signaling pathways, whereas CDV R252-infected cells showed much weaker expression of these pathways. These transcriptomic differences were further highlighted by the detection of the highest rates of cell apoptosis and lactate dehydrogenase activity in the supernatants of CDV Ond-infected AM cultures over the entire course of the experiment. In addition, transcriptome differences indicate disturbances of homeostatic AM functions associated with CDV infection. These results provide insights into early events in the pathogenesis of CDV infection and mechanisms underlying vaccine strain attenuation.

**IMPORTANCE:**

Morbilliviruses, including canine distemper virus (CDV) and human measles virus, cause severe systemic disease with respiratory distress, immunosuppression, and neurologic signs. While natural infection in dogs has become rare due to efficient vaccination, outbreaks in wildlife populations can be devastating, and concerns about zoonotic potential of CDV have been raised. The impact of CDV infection on the transcriptome of alveolar macrophages has not been elucidated thus far. Knowledge about early events in CDV pathogenesis and phenotypic consequences of vaccine attenuation is therefore necessary to protect endangered wildlife populations and might furthermore serve as a model for human measles. This study presents the first transcriptomic analyses of primary AMs during the initial phase of morbillivirus infection. These results provide insights into early events in the pathogenesis of CDV infection and mechanisms serving to restrict the spread of an attenuated virus strain.

## INTRODUCTION

Canine distemper virus (CDV, species *Morbillivirus canis*) is a member of the genus *Morbillivirus* within the family *Paramyxoviridae* (1). It causes canine distemper, a highly contagious and severe systemic disease in which respiratory distress, immunosuppression, and neurologic signs are typical clinical signs of infection. In addition to dogs, CDV infects wild carnivores, including seals, foxes, raccoons, bears, and mustelids, as well as large felids and non-human primates, thereby representing a serious threat to endangered wildlife species (2–5). Moreover, CDV outbreaks in rhesus and cynomolgus macaques in China and Japan, respectively, have raised concerns about the zoonotic potential of CDV, particularly due to decreasing human measles virus (MeV) vaccination rates in many regions (6–8). Live attenuated morbillivirus vaccines have been extremely successful in reducing morbidity and mortality levels in animals and humans in the last 60–70 years. However, our understanding of phenotypic differences between attenuated and wild-type strains in primary target cells is limited.

CDV has a non-segmented, negative sense, single-stranded RNA genome, six structural proteins, and two non-structural proteins (1). Morbillivirus non-structural V proteins interact with retinoic acid-inducible gene I (RIG-I)-like receptors, melanoma differentiation-associated protein 5 (MDA5), probable ATP-dependent RNA helicase DHX58, signal transducer and activator of transcription (STAT) 1, and STAT2, thereby inhibiting the production of type I interferons (IFNs) and tumor necrosis factor-α (TNF-α) (9–13).

A notable feature of morbilliviruses is their ability to infect epithelial cells of the respiratory tract, from which infectious virus is released and efficiently transmitted to other hosts via aerosols or respiratory droplets (14, 15). It is suspected that the initiation of CDV infection in a susceptible host is similar to MeV, bypassing the epithelial barrier of the respiratory tract via pulmonary dendritic cells and alveolar macrophages (AMs), which express the viral entry receptor CD150 (14, 16–18). Here, AMs are suggested to sustain primary respiratory MeV infection and are the immune cell type infected at the highest level in the lung of mice expressing human CD150 receptor during the early infection phase (16, 17). These infected cells subsequently transit the epithelial barrier of the respiratory tract with virus amplification occurring in lymphatic tissues of the respiratory tract, prior to the first viremic phase (16, 19, 20). The induction of timely and robust innate and humoral immune responses during early stages of infection in the respiratory tract could lead to a more restricted infection and effective virus elimination (21).

AMs are a distinct population of tissue resident macrophages originating from fetal progenitors (22, 23) and represent the first line of defense within lung alveoli, given the continuous exposure to infectious agents (24, 25). Under steady-state conditions, the functions of AMs include surfactant metabolism, phagocytosis, and clearance of cellular debris in order to maintain homeostasis within the lung microenvironment (26). Recognition of pathogen- or damage-associated molecular patterns by pattern recognition receptors and loss of their connection to epithelial cells in injured tissue can lead to a shift of AMs from a tolerogenic toward a pro-inflammatory phenotype, associated with the production of pro-inflammatory cytokines (27–31). AMs have been identified as early targets for MeV in transgenic mouse and cynomolgus monkey models, but there is a lack of knowledge about cellular responses upon infection (16, 17, 32). Their role as early target cells in CDV infection has also been suggested in ferret studies and *ex vivo* infection models (14, 33).

Modulation of innate immune cells provides a potential target for treatment and prophylactic approaches to mitigate the impact of viral diseases. However, knowledge about pulmonary innate immunity in morbillivirus infections and its impact on disease pathogenesis is still sparse. In particular, the transcriptional and phenotypic properties of AMs in canine distemper have not yet been investigated. Elucidating the regulatory mechanisms through which pathogens regulate innate immune cell plasticity will contribute to the discovery of therapeutic targets in morbillivirus-induced diseases and thus reduce virus transmission to other hosts. In this study, we have investigated the ability of a field and attenuated strain of CDV to productively infect primary canine AMs and show that this results in differential cytopathic effects and pro-inflammatory innate immune responses.

## RESULTS

### CDV infection of primary canine AMs is associated with a restricted infection by an attenuated strain

**Fig 1:**
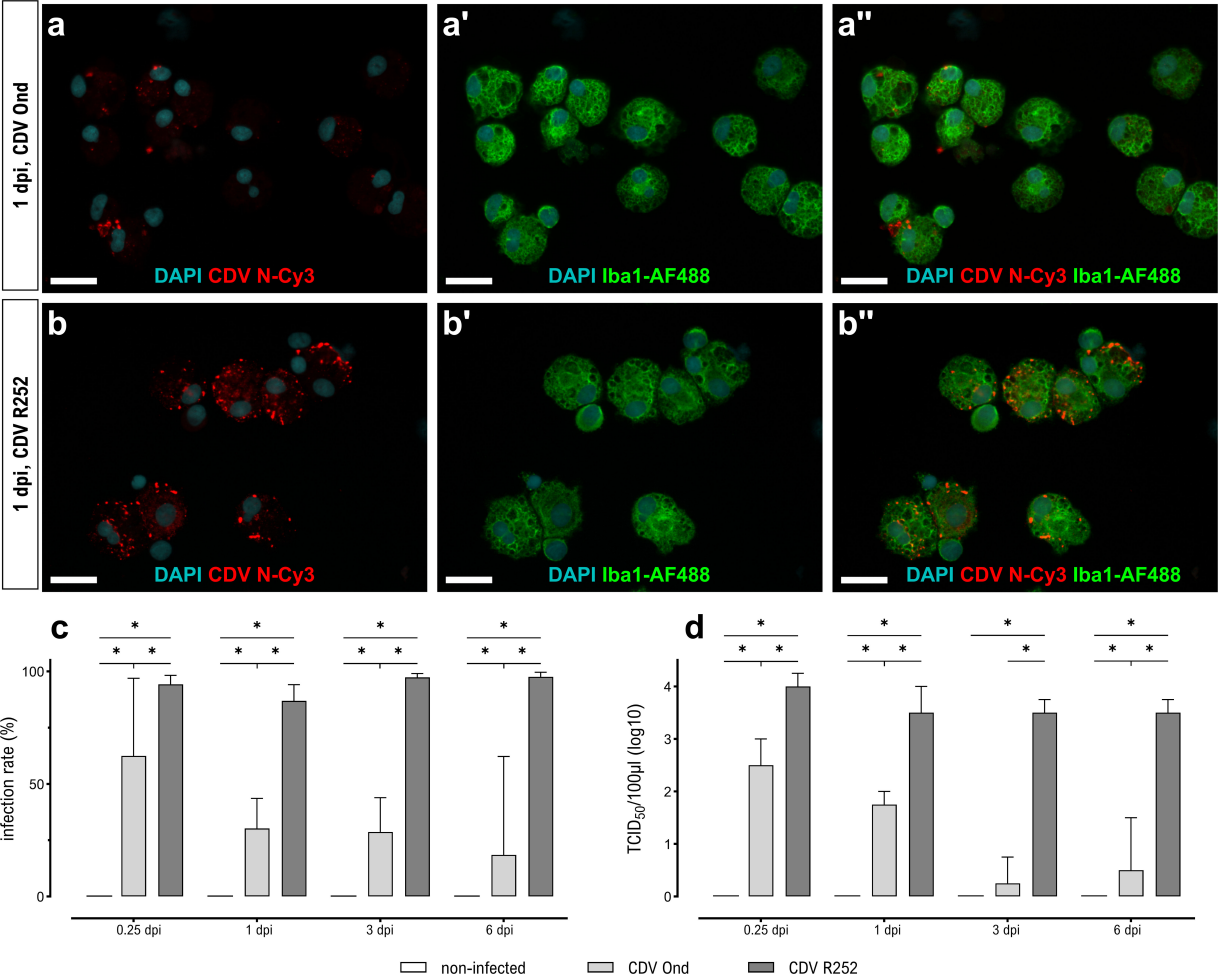
CDV infection of primary AMs. (**a and b**) Immunofluorescence staining of infected AMs for the detection of CDV nucleoprotein (N; red) and Iba1 (green). Nuclear counterstaining was performed using DAPI (blue). Scale bars = 20 µm. (**a**) AMs infected with CDV Ond at 1 dpi. (**b**) AMs infected with CDV R252 at 1 dpi. (**c**) Assessment of the virus infection rate via quantification of total numbers of CDV-N^+^/Iba1^+^ macrophages. (**d**) Quantification of virus production by TCID_50_ assay of cell culture supernatants. (**c and d**) Data are shown as median with 95% confidence interval. Significant changes are labeled by an asterisk (*P* ≤ 0.05, Mann–Whitney *U* test).

**Fig 2:**
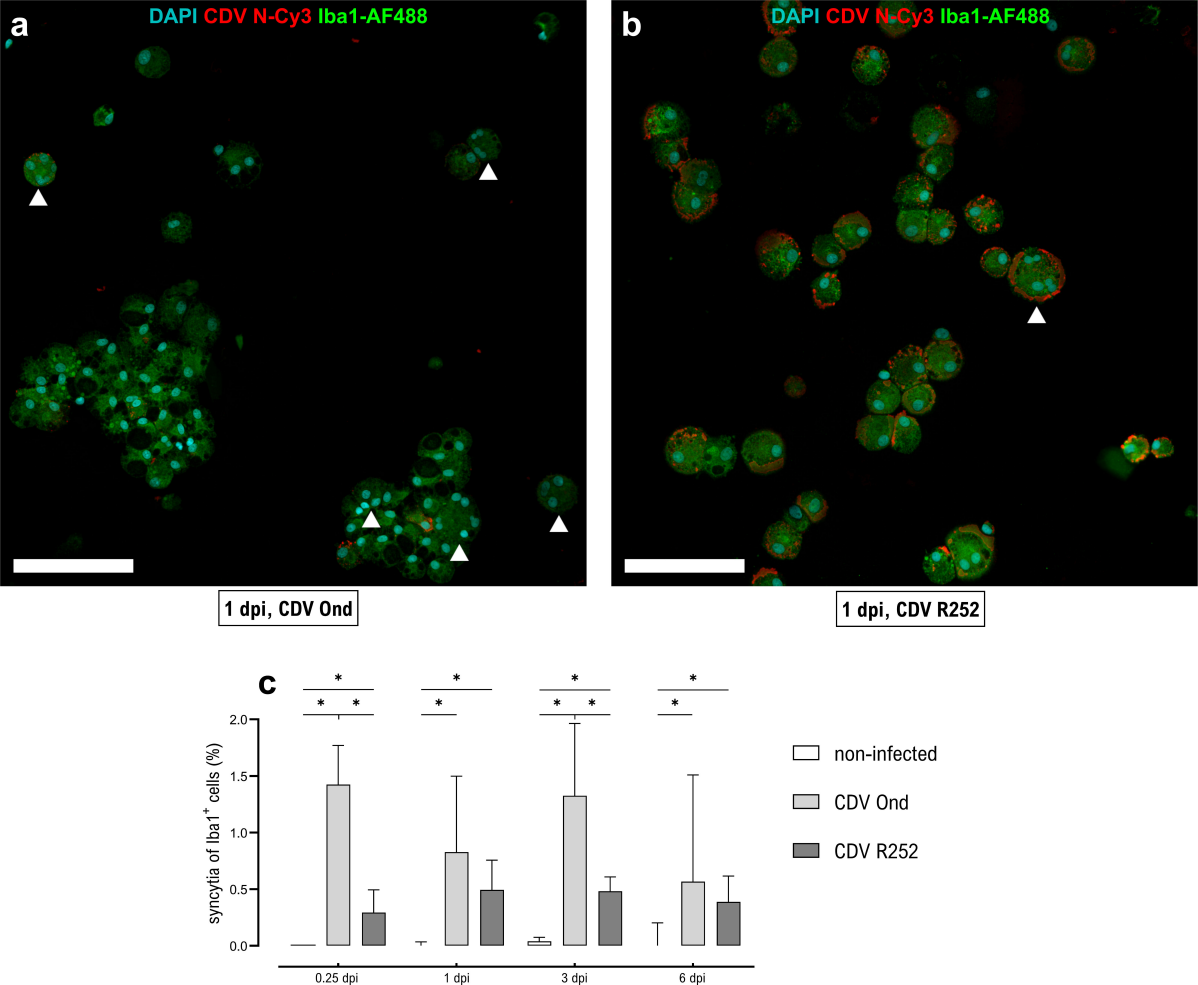
Syncytium formation observed in CDV-infected primary AMs cultures. (**a and b**) Immunofluorescence staining of AMs for detection of CDV nucleoprotein (N, red) and Iba1 (green). Nuclear counterstaining was performed using DAPI (blue). Scale bars = 200 µm. Syncytia with three or more nuclei are indicated by white arrowheads. (**a**) AMs infected with CDV Ond at 1 dpi. (**b**) AMs infected with CDV R252 at 1 dpi. (**c**) Quantification of syncytium formation of CDV-infected Iba1^+^ cells. Data are shown as median with 95% confidence interval. Significant changes are labeled by an asterisk (*P* ≤ 0.05, Mann–Whitney *U* test).

Productive infection with the attenuated Onderstepoort (Ond) or field R252 strains was confirmed by immunofluorescence staining (Fig. 1a through c) and virus titration at 6 hours, 1 day, 3 days, and 6 days post-infection (dpi) (Fig. 1d). Noteworthy, CDV R252 infected significantly higher numbers of Iba1^+^ AMs compared to the vaccine strain CDV Ond at all time points post-infection (Fig. 1c). Accordingly, a concomitant increase in the release of infectious viral particles was found in CDV R252-infected AMs at all time points (Fig. 1d). While a time-dependent decrease of infection rates and virus titers was observed in CDV Ond-infected cells, the number of infected cells and virus titers remained high in CDV R252-infected AM cultures. CDV-infected primary canine AMs showed prominent syncytium formation with significantly more multinucleated cells observed following CDV Ond infection in comparison to CDV R252-infected cells at all examined time points (Fig. 2a through c). However, much larger intracellular inclusions and aggregates of N protein were observed in CDV R252-infected AM cultures (Fig. 2a and b). These results show that canine AMs are permissive for CDV infection, but that viral replication and release of infectious virus particles is strain-dependent, suggesting differential antiviral responses and thus consequently different rates of viral elimination in infected cells. Correlating to these results *in vitro*, CDV antigen can be detected in alveolar histiocytes of naturally infected dogs. Immunohistochemistry and immunofluorescence double labeling were used to confirm natural CDV infection of Iba1^+^ AMs in lungs (Fig. S1a and b).

### AMs show enhanced cytokine responses following infection with an attenuated CDV strain

**Fig 3:**
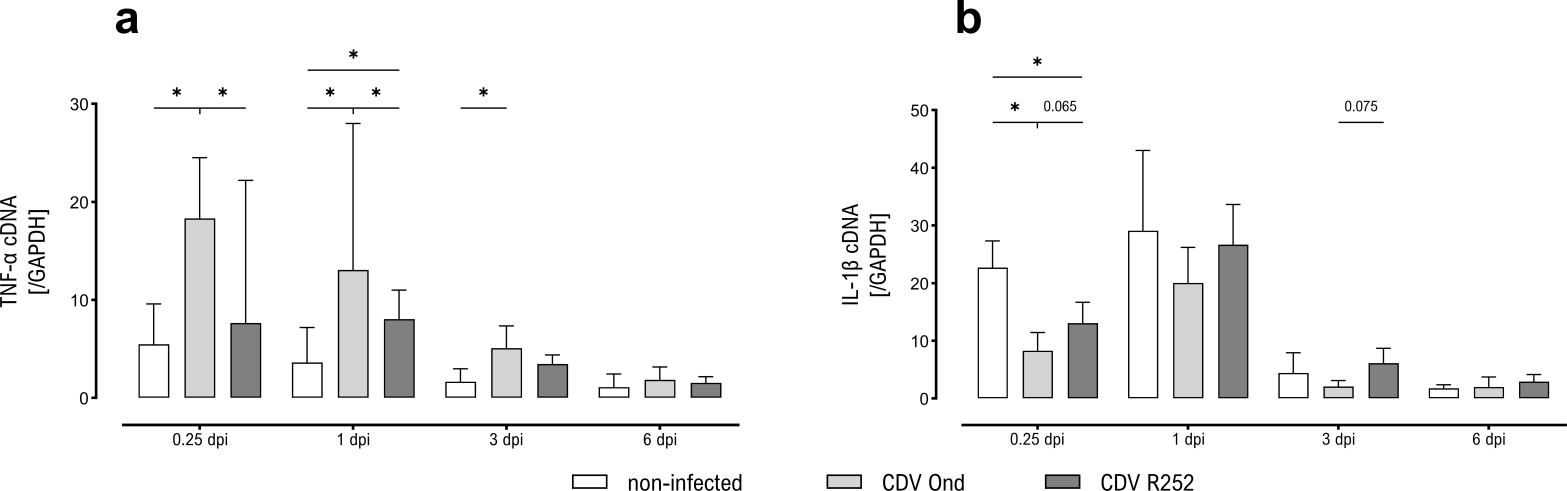
Cytokine expression of primary AMs infected with CDV Ond and R252 strains. (**a**) TNF-α mRNA expression, normalized by comparison to housekeeping gene GAPDH. (**b**) IL-1β mRNA expression, normalized by comparison to GAPDH. Data are shown as median with 95% confidence interval. Significant changes are labeled by an asterisk (*P* ≤ 0.05, Mann–Whitney *U* test).

To characterize cellular responses following *in vitro* CDV infection, cytokine expression of infected primary canine AMs at 6 hours, 1 dpi, 3 dpi, and 6 dpi was assessed by reverse transcription quantitative PCR (RT-qPCR). The transcription of TNF-α was most prominent in CDV Ond-infected samples with significantly increased levels compared to non-infected controls and CDV R252-infected cells at 6 hours pi and 1 dpi. In addition, a significant increase of TNF-α mRNA in CDV Ond-infected cells compared to controls was found at 3 dpi. Significantly higher expression of TNF-α mRNA in CDV R252-infected cells compared to non-infected AMs was found at 1 dpi (Fig. 3a). In contrast, significantly decreased interleukin (IL)-1β mRNA expression was found in both CDV Ond- and CDV R252-infected cells compared to non-infected samples at 6 hours pi. In addition, IL-1β mRNA expression in CDV Ond-infected cells showed a tendency toward lower expression compared to CDV R252-infected AMs (*P* = 0.065; Fig. 3b). Moreover, a statistical trend toward decreased IL-1β mRNA expression was found in CDV Ond-infected cells compared to R252-infected samples at 3 dpi (*P* = 0.075). Highest mRNA expression levels of other investigated cytokines were also found in CDV Ond-infected cells, peaking at 6 hours pi (IL-10 and IL-12) and 1 dpi (IL-6, IL-8, TGF-β, and IFN-γ), but group differences did not reach the levels of significance (Fig. S2).

### Transcriptome analyses reveal CDV strain-specific responses of AMs

**Fig 4:**
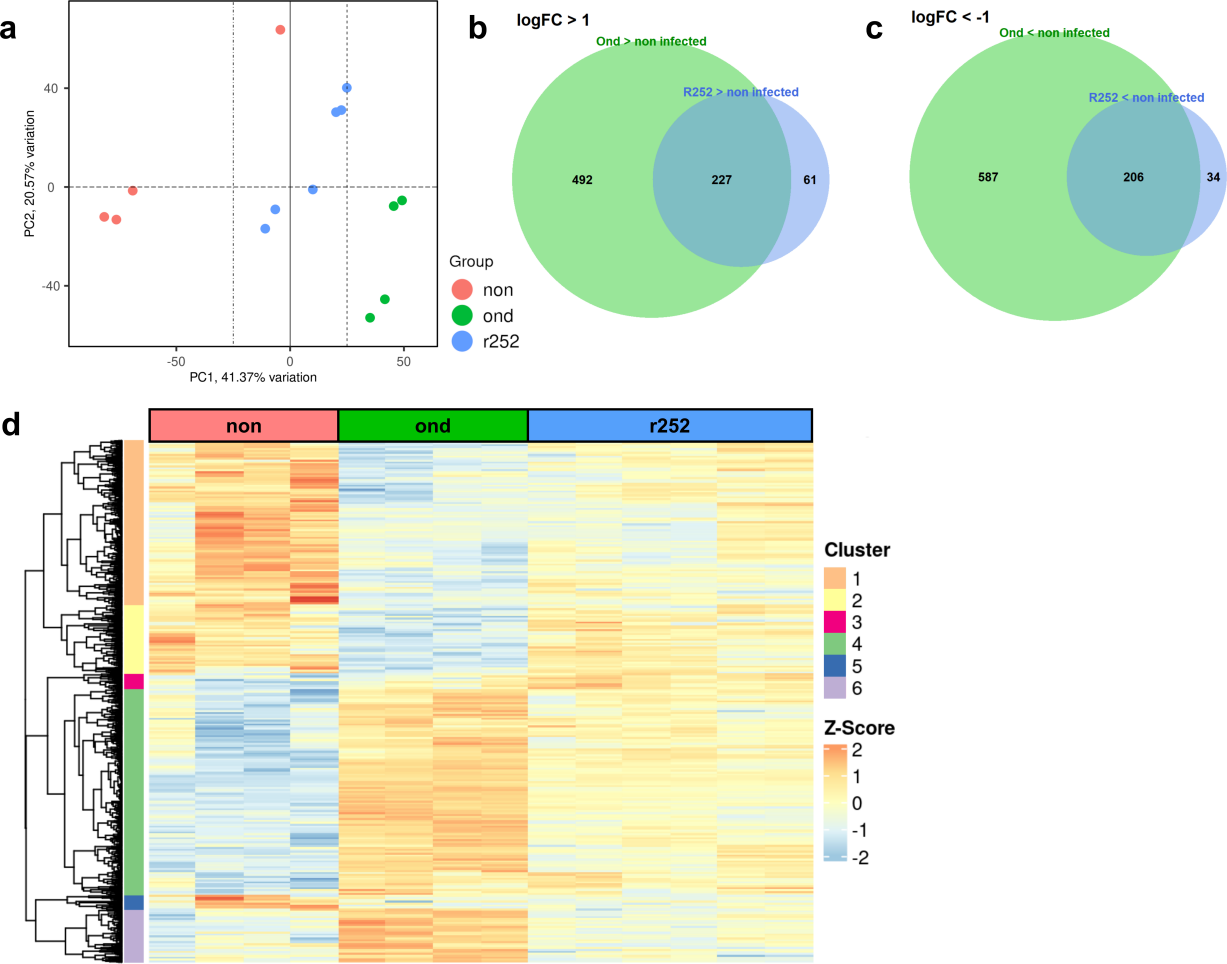
RNA-seq analyses of CDV-infected, primary AMs at 1 dpi. (**a**)Principal component analysis plot. Points indicate individual samples. Red circles represent non-infected samples, blue circles represent CDV R252-infected samples, and green circles represent CDV Ond-infected samples. (**b and c**) Venn diagrams: green areas indicate unique DEGs in CDV Ond-infected cells, blue areas indicate unique DEGs in CDV R252-infected cells, and overlay areas indicate shared DEGs. (**b**) Upregulated DEGs, log fold change (logFC) >1. (**c**) Downregulated DEGs, logFC − 1. (**d**) Heatmap of 1,662 DEGs in non-infected, CDV Ond, and CDV R252-infected AMs with logFC > 1 and adjusted *P* < 0.05.

In order to investigate the transcriptomic signature of canine AMs following CDV infection in more detail, total RNA sequencing (RNA-seq) of infected and non-infected samples during the early infection phase (1 dpi) was performed. Key functional differences were computed via principal component analysis, which clustered samples into three distinct subsets, corresponding with the group assignment (Fig. 4a). Pairwise comparison showed fundamental changes in the transcriptome, with higher numbers of up- or downregulated differentially expressed genes (DEGs) in canine AMs observed following CDV Ond infection compared to CDV R252 infection (Fig. 4b and c). In total, 492 genes were uniquely upregulated in CDV Ond-infected cells, 227 upregulated DEGs were shared in both CDV Ond- and CDV R252-infected samples, and 61 genes were upregulated solely in CDV R252-infected cells. For downregulated DEGs, 587 genes were exclusively downregulated in CDV Ond-infected cells, 206 genes were shared, and 34 genes were downregulated solely in CDV R252-infected samples. Hierarchical clustering based on expression profiles identified six different clusters (Fig. 4d; Table S1). DEGs in cluster 4 (*n* = 593) were upregulated in both CDV-infected groups, with significantly higher expression values in CDV Ond-infected AMs. Cluster 6 (*n* = 151) genes were almost exclusively upregulated in CDV-Ond infected AMs. DEGs in cluster 1 (*n* = 560), cluster 2 (*n* = 243), and cluster 5 (*n* = 52) were downregulated in both CDV-infected groups. Of note, CDV R252-infected samples often showed significantly higher expression levels of genes in cluster 1 and partly in cluster 2 compared to CDV Ond-infected samples. Cluster 3 contained 63 DEGs with higher expression levels in CDV-infected AMs compared to non-infected controls. Analysis of viral gene expression (N, P, M, F, H, and L genes) at 1 dpi did not reveal significant differences between both CDV strains (Fig. S3).

### Genes related to pro-inflammatory and antiviral responses are preferentially upregulated in AMs infected with an attenuated CDV strain

The differential expression profiles were further analyzed with respect to biological context by performing Gene Ontology (GO) enrichment analysis (“biological process”; Table S2) and Kyoto Encyclopedia of Genes and Genomes (KEGG) pathway analysis (Table S3). Most GO terms and KEGG pathways enriched in cluster 4 were related to innate immune processes and defense responses (Table 1).

**TABLE 1:** Selected enriched GO terms and KEGG pathways in clusters 1–6*^a^*

Gene set	GO terms (biological function)	Adjusted *P*-value	KEGG pathways	Adjusted *P*-value
DEGs in cluster 1 (*n* = 560) ▼∇^*b*^	Fatty acid catabolic process	0.000	Fatty acid metabolism	0.000
	Ribonucleotide biosynthetic process	0.003	Purine metabolism	0.050
DEGs in cluster 2 (*n* = 243) ▼∇^*b*^	Regulation of leukocyte-mediated immunity	0.016	Glycolysis/gluconeogenesis	0.027
	Actin cytoskeleton organization	0.046		
DEGs in cluster 3 (*n* = 63) ▲Δ	No enriched GO terms	–^*c*^	No enriched KEGG pathways	–^*c*^
DEGs in cluster 4 (*n* = 593) ▲^*b*^Δ	Defense response to virus	0.000	NF-kappa B signaling pathway	0.000
	Pattern recognition receptor signaling pathway	0.000	Apoptosis	0.000
	Type I IFN signaling pathway	0.001	Antigen processing and presentation	0.006
DEGs in cluster 5 (*n* = 52) ▼∇	Regulation of immune system process	0.019	Hematopoietic cell lineage	0.031
DEGs in cluster 6 (*n* = 151) ▲	No enriched GO terms		NF-kappa B signaling pathway	0.34
			NOD-like receptor signaling pathway	0.034

^*a*^ DEG = differentially expressed gene; IFN = interferon; NF-kappa B = nuclear factor kappa-light-chain-enhancer of activated B cells; NOD = nucleotide-binding oligomerization domain.▲: upregulated in CDV Ond-infected AMs compared to non-infected controls; ▼: downregulated in CDV Ond-infected AMs compared to non-infected controls; Δ: upregulated in CDV R252-infected AMs compared to non-infected controls; ∇: downregulated in CDV R252-infected AMs compared to non-infected controls.

^*b*^ Indicates the group with higher gene expression level.

^*c*^ –, not applicable.

**Fig 5:**
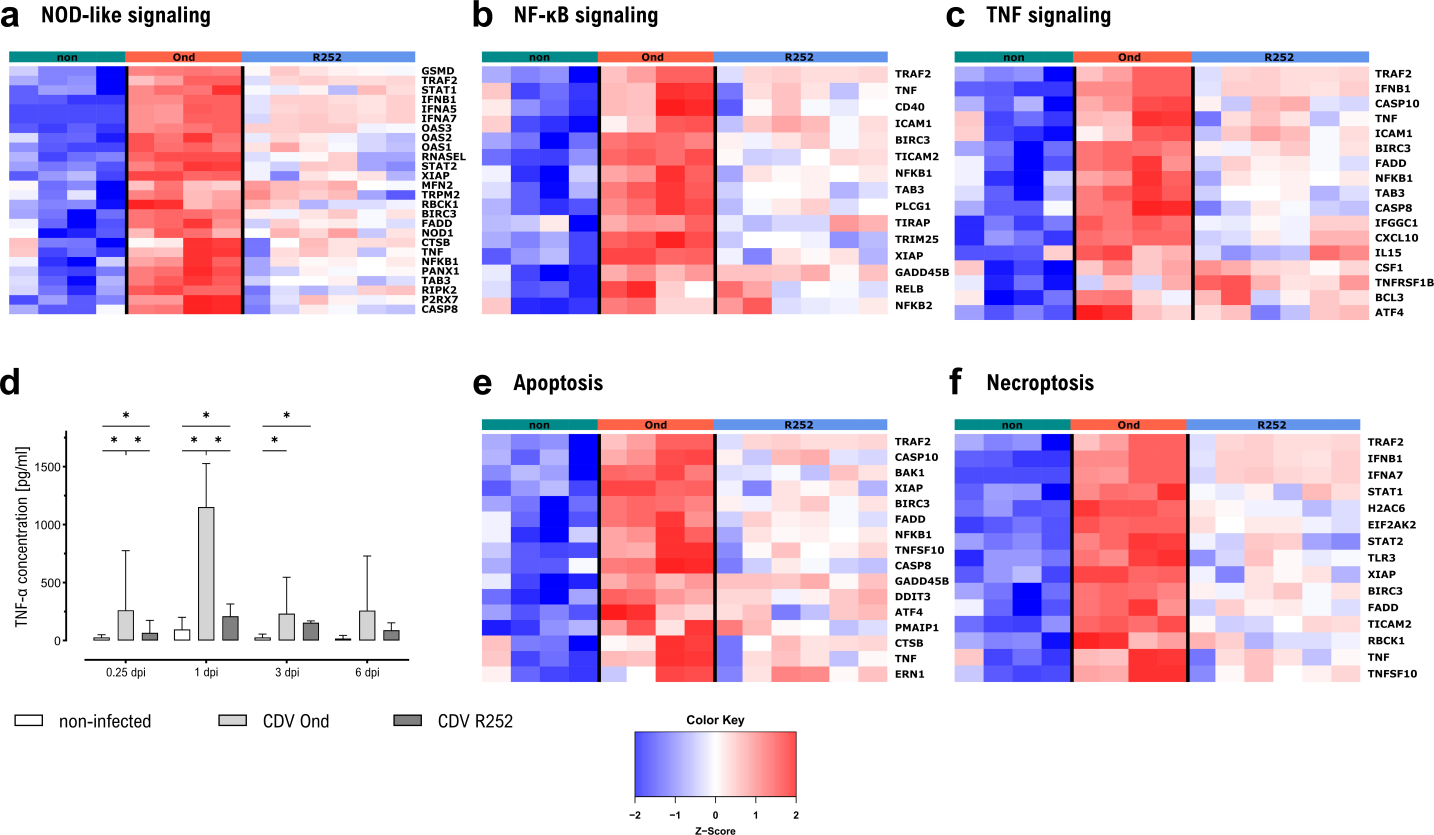
Selected GO terms and KEGG pathways related to pro-inflammatory and antiviral responses enriched in DEGs in cluster 4. (**a**) Heatmap showing expression of DEGs in cluster 4 in KEGG cfa04621 NOD-like receptor signaling pathway. (**b**) Heatmap showing expression of DEGs in cluster 4 in KEGG cfa04064 NF-kappa B signaling pathway. (**c**) Heatmap showing expression of DEGs in cluster 4 in KEGG cfa04668 TNF signaling pathway. (**d**) Concentrations of TNF-α in supernatants of CDV-infected canine AMs, measured by quantitative sandwich-ELISA. Data are shown as median with 95% confidence interval. Significant changes are labeled by an asterisk (*P* ≤ 0.05, Mann–Whitney *U* test). (**e**) Heatmap showing expression of DEGs in cluster 4 in KEGG cfa04219 apoptosis. (**f**) Heatmap showing expression of DEGs in cluster 4 in KEGG cfa04217 necroptosis.

KEGG pathway analysis revealed enrichment of several pathways associated with sensing of and reaction to pathogens in cluster 4, including NOD-like receptor signaling pathway (Fig. 5a), Toll-like receptor signaling pathway, and RIG-I-like receptor signaling pathway. Initiation of pro-inflammatory reactions to CDV infection was reflected by the enrichment of several GO terms and KEGG pathways associated with responses to virus infection, including the NF-κB signaling pathway, which is associated with the induction of pro-inflammatory components in immune cells and initiating antiviral defense responses (34) (Fig. 5b).

In agreement with RT-qPCR results (Fig. 3a), RNA-seq analysis revealed increased TNF-α gene expression in CDV Ond-infected canine AMs, while no upregulation of TNF-α was observed in CDV R252-infected canine AMs. Moreover, transcription of TNF-α -related genes was primarily upregulated in CDV Ond-infected cells (Fig. 5c). Similar to TNF-α mRNA expression (Fig. 3b), secretion of TNF-α by CDV Ond-infected AMs was significantly higher compared to both CDV R252- and non-infected AMs at 6 hours pi and 1 dpi, as shown by quantitative enzyme-linked immunosorbent assay (ELISA). TNF-α secretion by both CDV-infected AM cultures was significantly higher compared to the non-infected AMs at 6 hours pi, 1 dpi, and 3 dpi, with highest values found at 1 dpi (Fig. 5d). In addition, genes enriched in KEGG pathways apoptosis and necroptosis and GO terms associated with cell death were also present in cluster 4 (Fig. 5d and e). Interestingly, *CASP8* and *FADD*, associated with the initiation of the extrinsic apoptosis pathway by TNF-α, were only significantly higher expressed in CDV Ond-infected AMs compared to controls and CDV R252-infected AMs, while there was no upregulation of expression of these genes in CDV R252-infected AMs.

**Fig 6:**
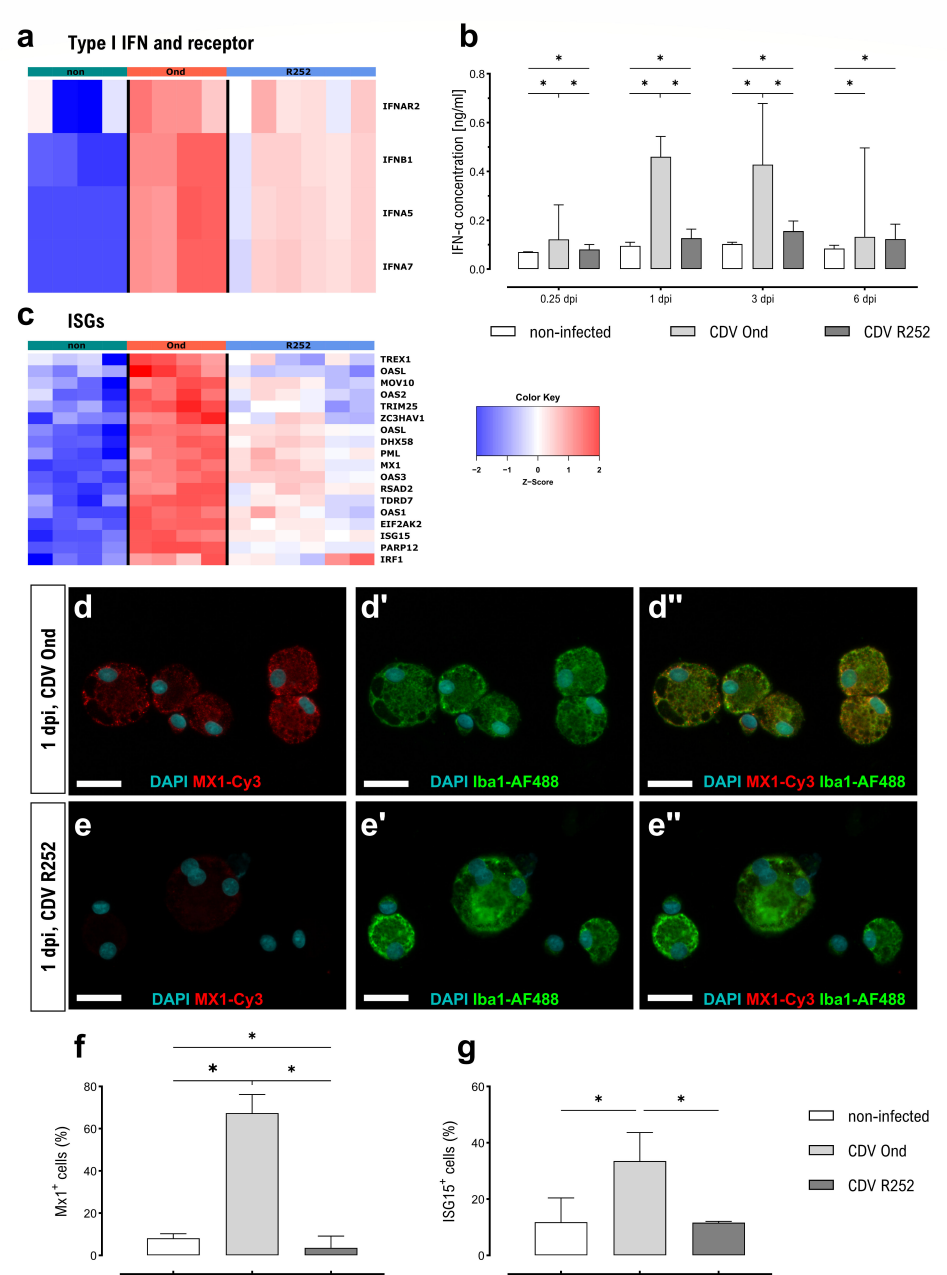
Type I IFN signaling in CDV-infected primary canine AMs. (**a**) Heatmap showing expression of genes encoding for type I IFN and their receptor. (**b**) Concentrations of IFN-α in supernatants of CDV-infected canine AMs, measured by quantitative sandwich-ELISA. (**c**) Heatmap showing expression of selected ISGs in cluster 4. (**d and e**) Immunofluorescence staining for detection of MX1 expression (red) in Iba1^+^ AMs (green). Nuclear counterstaining was performed using DAPI (blue). Scale bars = 20 µm. (**d**) AMs infected with CDV Ond at 1 dpi. (**e**) AMs infected with CDV R252 at 1 dpi. (**f**) Quantification of MX1 expressing Iba1^+^cells. (**g**) Quantification of ISG15 expressing Iba1^+^cells. (**b, f–g**) Data are shown as median with 95% CI. Significant changes are labeled by an asterisk (*P* ≤ 0.05, Mann–Whitney *U* test).

CDV infection led to an upregulated transcription of IFN-α and IFN-β genes as well as their receptor and downstream transcription factor *STAT1* in canine AMs. Increased transcription of the IFN-α gene *IFNA5* (logFC 15) and IFN-β gene *IFNB1* (logFC 11.5) was most prominent in CDV Ond-infected cells (Fig. 6a). Quantitative ELISA also revealed significantly increased IFN-α secretions by both CDV Ond- and R252-infected AMs compared to non-infected controls at all investigated time points. Significantly higher IFN-α secretion by CDV Ond-infected AMs compared to CDV R252-infected AMs was detected at 6 hours pi, 1 dpi, and 3 dpi (Fig. 6b).

Other cluster 4 genes, for which increased transcription is triggered by type I IFNs (IFN-stimulated genes [ISGs]) (35, 36) are presented in Fig. 6c. These genes were all upregulated in both CDV Ond- and CDV R252-infected AMs but most prominently in CDV Ond-infected AMs. These findings were subsequently confirmed on a protein level, by performing immunofluorescence staining of two selected ISG proteins (ISG15 and MX1) in infected AM cultures (Fig. 6d and e). In accordance with transcriptome analysis, the numbers of both ISG^+^ Iba1^+^ cells and MX1^+^ Iba1^+^ cells at 1 dpi were highest in CDV Ond-infected AM cultures compared to CDV R252-infected cells and non-infected controls. In addition, MX1 protein expression in CDV R252-infected AMs was significantly lower compared to non-infected cells (Fig. 6f and g).

Along with type I IFN and TNF-α signaling, several genes encoding for chemokines were upregulated as parts of clusters 4 and 6. While CDV Ond-infected AMs showed a significant upregulation of *CXCL10*, *CCL8*, *CCRL2*, *CCL5*, and *CCL4* compared to non-infected controls, only *CXCL10* was upregulated in CDV R252-infected cells.

KEGG pathway and the eponymous GO term antigen processing and presentation were enriched in cluster 4 and upregulated in CDV-infected cells, containing genes related to antigen processing and presentation via MHC peptides. All genes related to antigen presentation were slightly upregulated (logFC < 2.5), and for most genes, differential expression was significant only between CDV Ond-infected cells and non-infected controls.

### Attenuation of CDV is associated with increased cytopathic effects in infected AM cultures

**Fig 7:**
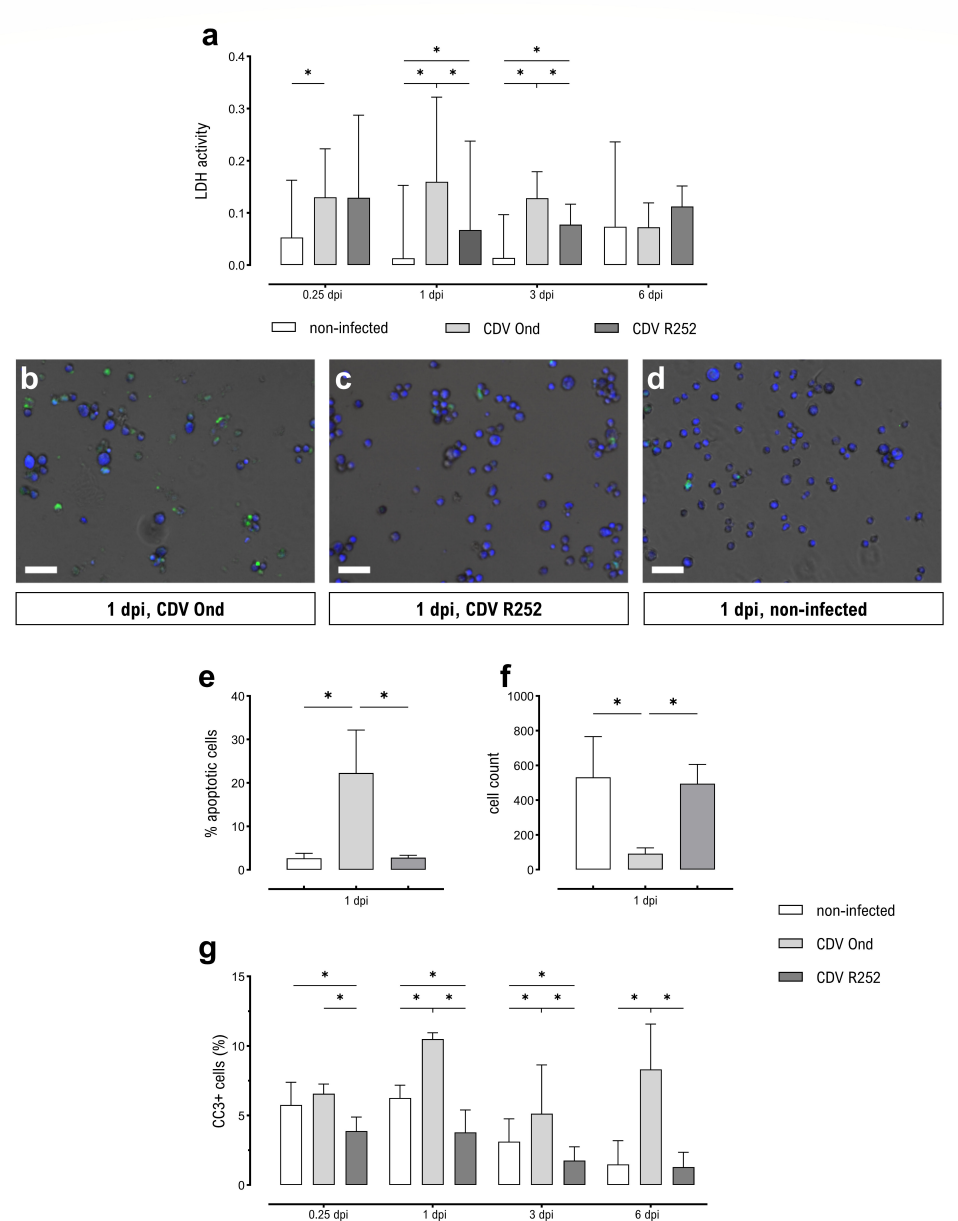
Cytopathic effects in CDV-infected primary AMs. (**a**) LDH activity in supernatants of CDV-infected AMs. (**b–d**) Representative micrographs showing intact (CytoCalcein Violet 450^+^, blue) and apoptotic (Apopxin Green^+^, green) cells, overlayed with phase contrast images. Scale bars = 50 µm. (**b**) AMs infected with CDV Ond at 1 dpi. (**c**) AMs infected with CDV R252 at 1 dpi. (**d**) Non-infected AMs at 1 dpi. (**e**) Quantification of apoptotic cells at 1 dpi. (**f**) Cell counts of CDV-infected AMs at 1 dpi. (**a, e–g**) Data are shown as median with 95% CI. Significant changes are labeled by an asterisk (*P* ≤ 0.05, Mann–Whitney *U* test).

Both the observation of syncytium formation and the enrichment of cell death pathways in cluster 4 are indicative of increased cytopathic effects in AMs following CDV infection. To further evaluate the cell death of CDV-infected primary canine AMs, lactate dehydrogenase (LDH) activity was measured in supernatants collected at 6 hours pi, 1 dpi, 3 dpi, and 6 dpi (Fig. 7a). At 6 hours pi, LDH release by CDV Ond-infected AMs was significantly higher compared to non-infected AMs, indicating reduced cell viability. At 1 and 3 dpi, LDH release by CDV Ond-infected AMs peaked and was significantly higher compared to both other groups of AMs. Also, CDV R252-infected AMs released significantly more LDH compared to non-infected control AMs at 1 and 3 dpi.

Further insights into different cell death pathways were obtained by differential cell staining to distinguish between live, apoptotic, and necrotic cells at 1 dpi (Fig. 7b through d). While the number of necrotic cells was negligible (<0.5%, data not shown), apoptotic cell counts showed significant differences between the three groups. While no differences were found between CDV R252-infected cells and non-infected controls and rates of apoptosis were below 4.5%, markedly higher rates of apoptosis up to 36% were observed in CDV Ond-infected cells (Fig. 7e). Corresponding with both increased LDH activity and apoptotic cell count in CDV Ond-infected cells, live cell counts were lower than in both other groups (Fig. 7f).

To examine apoptosis induction in CDV-infected AMs over the entire course of the experiment, immunofluorescence staining of cleaved caspase-3 (CC3) was performed (Fig. 7g). Here, CDV Ond-infected AMs showed a higher proportion of CC3^+^ cells at 6 hours pi compared to CDV R252-infected cells and at 1, 3, and 6 dpi compared to both other groups. The number of CC3^+^ AMs peaked at 1 dpi. Interestingly, at 6 hours pi, 1 dpi, and 3 dpi, the percentage of CC3^+^ AMs was significantly lower in CDV R252-infected AMs compared to non-infected AMs. Taken together, this suggests that CDV Ond-infection is associated with reduced cell viability and enhanced apoptosis of canine AMs.

### CDV infection of AMs downregulates genes related to cell homeostasis and immune cell interaction

Analysis of cluster 2 identified genes, which were downregulated in both groups of CDV-infected cells compared to non-infected AMs, including genes related to phagocytosis, cell adhesion, migration, and cell-cell interaction. Genes related to the GO terms regulation of leukocyte-mediated immunity, regulation of immune effector process, and T cell-mediated immunity were associated with pro-inflammatory signaling (*IL1B*), leukocyte adhesion (*FUT7* and *ITGAM*) (37, 38), phagocytosis of apoptotic cells (efferocytosis) (*RAC2* and *ITGAM*) (39, 40), and reactive oxygen species generation (*RAC2*) (41) (Fig. S4a). Furthermore, genes related to the GO term actin cytoskeletal organization were part of cluster 2 and significantly downregulated in CDV Ond-infected samples (Fig. S4b). Organization of the cell cytoskeleton is a key component of phagocytic function. In addition, cluster 1 contained *CD36*, a macrophage scavenger receptor involved in the recognition of bacterial pathogens and phagocytosis of apoptotic cells (42–44), which was downregulated in both infected groups. This clearly indicates that pro-inflammatory responses, which were pronounced in CDV Ond-infected cells, were accompanied by the downregulation of genes related to homeostatic functions.

### CDV infection alters the transcription of metabolic genes in AMs

The majority of enriched KEGG pathways and GO terms within cluster 1 were associated with cellular metabolism and downregulated in CDV-infected AMs. Promoters of fatty acid oxidation *CD36* and *IRF4* (45–47) showed highest expression in non-infected samples and were downregulated predominantly in CDV Ond-infected samples. The KEGG pathway fatty acid metabolism contained genes associated with peroxisomal and mitochondrial fatty acid β-oxidation (Fig. S4c). In addition, several GO terms and KEGG pathways associated with nucleotide metabolism were enriched in cluster 1 (ribonucleotide biosynthetic process, purine nucleotide biosynthetic process, and purine metabolism) (Fig. S4d). DEGs of the GO term ribonucleotide biosynthetic process were downregulated predominately in CDV Ond-infected AMs. Furthermore, genes enriched in the KEGG pathway purine metabolism, including genes encoding for enzymes involved in *de novo* synthesis of purines, were significantly downregulated in CDV-Ond-infected samples compared to non-infected controls. Collectively, transcriptome analyses from the initial infection phase revealed alterations of cellular lipid and nucleotide metabolism in AMs following CDV infection, suggestive of a shift from AM homeostatic fatty acid oxidation toward a more glycolytic energy generation, which is associated with the pro-inflammatory polarization of macrophages.

## DISCUSSION

Morbilliviruses are highly contagious due to efficient spread via the respiratory route with AMs and dendritic cells identified as the primary target cells during early stages of infection in the respiratory tract (16, 17, 32). Similarly, infection of Iba1^+^ macrophages within alveoli and associated expression of TNF-α and ISG proteins has been reported in dogs naturally infected with CDV (48). This study shows higher infection rates and virus titers in primary canine AMs following infection with the field CDV R252 strain compared to the CDV Ond vaccine strain over the entire course of the experiment. Moreover, accelerated virus elimination in CDV Ond-infected AMs is indicative of a more robust and efficient innate immune response. Accordingly, transcriptome analyses at one dpi, representing the initial infection phase, revealed increased gene expression associated with pro-inflammatory pathways and type I IFN signaling in AMs infected with the attenuated virus strain. These findings highlight the phenotypic consequences of morbillivirus strain attenuation in a primary cell model in which virus spread is restricted in comparison to a field isolate.

ISGs, found to be upregulated in CDV-infected AMs, encode for multiple proteins, which have been shown to restrict virus replication in different stages of the viral life cycle. This includes transcriptional elongation (MX1) (49), inhibition of the production of viral proteins (PKR, encoded by *EIF2AK2*) (50), interference with viral replication (viperin, encoded by *RSAD2*) (51), and the degradation of viral RNA via OAS proteins that activate RNase L (encoded by *RNASEL*) (52). Moreover, type I IFNs have the potential to increase virus-induced apoptosis (53). Immunofluorescence staining showed that MX1 and ISG15 proteins are expressed prominently in AMs infected with the attenuated CDV Ond strain. Thus, enhanced expression of ISGs could contribute to viral elimination observed in CDV Ond-infected AM cultures, while less efficient antiviral signaling by AMs during initial CDV R252 infection might facilitate prolonged virus infection, as reflected by continuously high infection rates and virus titers over the 6-day course of the experiment.

In viral diseases, apoptosis is a basic mechanism to limit the extent of viral replication and cell-to-cell spread (54, 55). Wild-type CDV has been shown to prevent apoptosis of infected kidney epithelial cells *in vitro* and of infected immune cells of experimentally infected ferrets *in vivo*, representing a possible mechanism of immune evasion (56). In contrast to wild-type CDV strains, infection of Vero cells with CDV Ond causes caspase-3- and caspase-8-mediated apoptosis (57). TNF-α gene expression and protein secretion were increased primarily in CDV Ond-infected AMs but only slightly in CDV R252-infected AMs, indicating an insufficient cytokine response in the latter. Enhanced TNF-α expression in CDV Ond-infected AMs might thus contribute to reduced viral replication and prevention of viral spread by its function as an inducer of cell death. This is supported by the upregulation of apoptosis-related genes during initial infection and high LDH release and increased apoptosis rates in these CDV Ond-infected AM cultures. Suppressed TNF-α expression in peripheral blood mononuclear cells from experimentally CDV-infected ferrets has been previously observed: while TNF-α mRNA expression is downregulated in blood leukocytes from virulent CDV-infected ferrets, V protein-knockout recombinant CDV induces increased TNF-α mRNA expression (9). Thus, enhanced cell death of primary canine AMs following CDV Ond-infection is probably associated with virus attenuation, leading to more rapid viral clearance. In the present study, CDV R252-infected AMs maintained high infection rates and production of infective virus particles over the entire course of the experiment, accompanied by low apoptosis rates. Thus, field CDV strains such as R252 might inhibit apoptosis of primary target cells, which could contribute to viral immune evasion and increased spread (56). The enhanced type I IFN and TNF-α signaling in CDV Ond-infected AMs contributes to the activation of death signaling cascades of infected cells and thus efficient elimination of virus-infected cells (58–60). Given the essential role of early pro-inflammatory responses of AMs in other respiratory viral infections, including influenza A virus and respiratory syncytial virus, an insufficient response and lack of cell death during aerogenic CDV infection might promote early viral propagation in AMs and spread to other cells of the respiratory tract (61, 62).

Another function of IFN signaling is the initiation of antigen presentation to induce adaptive T-cell responses (63, 64). To maintain tolerance to innocuous antigens within the alveolar niche, AMs are relatively poor antigen presenters and can actively suppress T-cell activation by dendritic cells under homeostatic conditions (65–67). CDV Ond-infected AMs showed increased expression of genes associated with the immunoproteasome and loading of MHC class I molecules and genes related to MHC class II-mediated antigen presentation, probably as a consequence of type I IFN signaling (63, 64). In addition, the enhanced chemokine response in CDV Ond-infected cells corresponds well with the higher transcription of genes related to type I IFN and TNF-α signaling. Chemoattraction of inflammatory cells is necessary to create an antiviral microenvironment and initiate adaptive immunity (65, 68). Therefore, the lack of chemokine induction together with a disturbed antigen presentation capacity in CDV R252-infected AMs might facilitate virulence and impair antiviral immunity.

The *IL1B* gene was significantly downregulated preferentially in CDV Ond-infected AMs as shown by RT-qPCR and RNA-seq analyses of the AMs during the initial infection phase. Type I IFN signaling decreases the amount of IL-1β, since type I IFN treatment of human macrophages suppresses pro-IL-1β protein availability, its caspase-1-mediated cleavage, and the release of IL-1β (69, 70). Similarly, human metapneumovirus suppresses IL-1β, associated with IFN-β signaling in human monocytes *in vitro* (71). Hence, type I IFN responses in CDV Ond-infected AMs and to a lesser extent in CDV R252-infected AMs may account for the downregulation of *IL1B* expression found in the current study.

Formation of syncytia is a common finding in lungs of CDV-infected dogs (48). CDV Ond infection was observed to cause more extensive cytopathic effect in infected AMs, as evidenced by increased numbers of syncytia. Increased syncytium formation in Vero cells is a characteristic feature of this attenuated strain and is related to properties of the H protein (72). *In vitro* studies on MeV-infected human epithelial and dendritic cells revealed a correlation between cell fusion with the consequent formation of multinucleated cells and increased cellular IFN-β production, as is also observed in the current study (73).

Viruses benefit from faster levels of replication, which involves active nucleotide metabolism (74). Several IFN-regulated mechanisms are known to disturb nucleotide metabolism, which has been shown to restrict lentivirus and herpesvirus infections. Genes related to nucleotide metabolism were downregulated in AMs infected with both CDV strains but more pronounced in CDV Ond-infected AMs, possibly representing another antiviral mechanism during the initial phase of CDV infection.

AMs exhibit several important regulatory functions, protecting the lung environment from harmful overreactions of the immune system in response to inhaled particles. Migration along the respiratory epithelium, phagocytosis of inhaled particles, scavenging microbes, clearance of apoptotic cells (efferocytosis), and maintenance of pulmonary surfactant homeostasis are all essential tasks of AMs within their anatomical niche (24, 75). Under physiologic conditions, AMs have a low rate of glycolysis and a dominance of energy metabolism utilizing lipids (76). Non-infected AMs showed the highest expression of genes related to fatty acid catabolism, which is essential to remove excess surfactant and a hallmark of anti-inflammatory M2-polarized macrophages (77). Genes associated with fatty acid metabolism were downregulated early during CDV infection, which was most pronounced in CDV Ond-infected cells. This downregulation in CDV-infected cells might indicate a metabolic switch to a pro-inflammatory and glycolytic cell type, representing a downstream effect of Toll-like receptor signaling (78). Downregulation of *IDH1* (encoding for isocitrate dehydrogenase) implies a metabolic switch and M1 polarization of CDV-infected AMs (79). Disruption of homeostatic AM functions during initial CDV infection could predispose infected animals to secondary bacterial pneumonia, as shown in respiratory syncytial virus and murine cytomegalovirus infection of mice (80, 81).

The V protein of wild-type morbillivirus strains is able to block antiviral responses by inhibiting the translocation of transcription factors STAT1 and STAT2 to the nucleus (10, 82, 83). Moreover, the interaction of the V protein with RIG-I-like receptors MDA5 and LGP2 directly interferes with IFN-β transcription (11, 13, 84–87). Thus, the transcriptional differences observed between CDV R252- and CDV Ond-infected AMs could be due to a change in the V protein of CDV Ond affecting STAT1 translocation to the nucleus (10, 88). Given that morbillivirus attenuation occurs due to mutations of several genes (89), future studies could use the AM model to better delineate the contribution of specific viral molecular determinants to observe transcriptional and phenotypic differences in CDV Ond- and CDV R252-infected AM cultures.

In summary, the present study shows that primary canine AMs can be efficiently and productively infected by CDV. Virus infection induces pro-inflammatory innate immune responses during the initial infection phase, which is dominated by type I IFN signaling. Comparison of virus strains reveals enhanced pro-inflammatory signaling and cytotoxic effects in AMs infected with the attenuated strain of CDV, suggestive of an insufficient induction of antiviral pathways by CDV R252. Despite the induction of a highly activated, antiviral transcriptional signature, CDV infection also induced changes in gene expression associated with homeostatic processes of AMs. Disturbance of cellular metabolism and reduced ability to clear apoptotic cells from the alveolar microenvironment might represent factors facilitating secondary bacterial infections upon natural CDV infection. Thus, this study not only identifies AMs as target cells of CDV and confirms previously suggested mechanisms of viral interference with innate immune signaling but also highlights functional disturbances of key AM functions.

A limitation of the current study is the performance of bulk RNA-seq solely at the early initial phase of infection (1 dpi). Therefore, the temporal development of antiviral responses in CDV-infected AMs could not be monitored. In addition, further experiments, including functional assays, are clearly needed to get in-depth insights into disturbed homeostatic AM functions and cell metabolism during morbillivirus infection, as indicated by transcriptomic alterations.

## MATERIALS AND METHODS

### Isolation and culture of primary canine AMs

Isolation of AMs was performed according to a protocol published by Busch and co-workers with slight modifications (90). In brief, AMs were isolated from bronchoalveolar lavage (BAL) fluid of 11 recently deceased dogs. The authors confirm that no animals were sacrificed for the purpose of this study. All dogs used in the present study were dead at the time of submission to routine necropsy service following euthanasia due to animal welfare reasons. Owners declared written consent for sample collection. Histopathologic examination of lung tissue performed by European College of Veterinary Pathologists board-certified veterinary pathologists (AB, MS) ruled out respiratory disease and evidence for viral, bacterial, mycotic, or parasitic infections of the respiratory tract. The collection of BAL was performed using pre-warmed (37°C) phosphate-buffered saline (PBS)-based buffer containing 2 mM EDTA (Biochrom) and 0.5% (vol/vol) fetal bovine serum (Capricorn Scientific). The lavage fluid was diluted, filtered through a 100 µm cell sieve, and centrifuged. Red blood cell lysis was performed by incubating cell pellets for 5 minutes with 10% lysis buffer (0.155 M NH_4_Cl, 0.01 M KHCO_3_, and 0.1 M EDTA), and cells were centrifuged and filtered through a 40 µm cell sieve twice. Cells were seeded at a maximum density of 5 × 10^7^ cells per T75-flask in Roswell Park Memorial Institute (RPMI)-medium 1640 with L-alanyl-glutamine and sodium bicarbonate (Thermo Fisher Scientific), supplemented with 1% (wt/vol) sodium pyruvate (Sigma-Aldrich), 1% (vol/vol) penicillin/streptomycin (Sigma-Aldrich), and 10% (vol/vol) fetal bovine serum. After overnight incubation at 37°C with 5% CO_2_, cells were carefully collected from the flasks with a cell scraper and seeded at a density of 1 × 10^5^ cells per well in a 24-well plate (Sarstedt) for use in virus infection experiments. Infection was performed 1 day after cell culture preparation in order to prevent cellular adaptation to culture conditions (91). Immunofluorescence staining for Iba1 revealed a purity of histiocytic cells of over 90% (median).

### Cell lines

DH82 cells were obtained from the European Collection of Authenticated Cell Cultures (ECACC No. 94062922) and used as a control for immunofluorescence staining. This cell line originates from a malignant histiocytosis of a dog (92). The derivation of DH82 cells persistently infected with the Ond strain of CDV was performed as previously described (93). Cells were cultured in Minimal Essential Medium with Earle’s salts (Thermo Fisher Scientific) with 10% (vol/vol) fetal bovine serum, 1% (vol/vol) penicillin/streptomycin, and 1% (vol/vol) non-essential amino acids (Sigma-Aldrich). Vero cells stably expressing canine SLAM (Vero-SLAM cells) (94) were used to determine the tissue culture infectious dose−50 (TCID_50_) of virus present in collected supernatants. Vero-SLAM cells were cultured in Dulbecco’s Modified Eagle’s medium (Thermo Fisher Scientific) containing 1% (vol/vol) penicillin/streptomycin, 10% fetal bovine serum, and 0.5 mg/mL Zeocin (InvivoGen).

### Viruses

Two different strains of CDV were used for infection. CDV R252 was isolated from spleen tissue of a naturally infected dog (kindly provided by Prof. S. Krakowka, Ohio State University, Columbus, OH, USA). The strain was shown to cause lethal infection with severe pathology of the lymphoid and/or central nervous system in gnotobiotic dogs and ferrets, respectively, and to induce cytopathic effects in primary canine brain cell cultures (95–98). CDV R252 was propagated in Vero cells, reaching a titer of 10^6.5^ TCID_50_/mL. CDV Ond was first isolated in 1939 following an outbreak of canine distemper in North American ranched foxes and has undergone multiple passages in ferrets and eggs (99). The used strain originates from the Belfast variant of CDV Ond, harboring the Y110D mutation in the V protein (10). It was propagated in Vero cells and attained a titer of 10^6^ TCID_50_/mL.

### Virus infection of primary cultures

Prior to infection, cells were washed twice in cell culture medium devoid of serum (washing medium), followed by incubation with washing medium containing virus inoculum to enable infection at a multiplicity of infection of 1. After 3 hours of incubation at 37°C and 5% CO_2_, washing medium was replaced by culture medium and cells were incubated until harvesting at 6 hours pi, 1 dpi, 3 dpi, and 6 dpi. Cells were carefully detached from the wells using a cell scraper (Sarstedt), and an aliquot was taken for Cytospin centrifugation. A centrifugation step was performed to remove the supernatant, and both supernatants and cell pellets were rapidly frozen in liquid nitrogen and stored at −80°C until further use.

### Virus titration

To assess viral loads within supernatant, a TCID_50_ assay was performed. Therefore, 3 × 10^4^ Vero-SLAM cells were seeded in each well of a 96-well plate in 100 µL growth medium. Supernatants obtained from infection experiments were serially diluted in washing medium with four replicates per dilution and medium-only controls. After 6 days, the presence of cytopathic effects in each well was assessed by light microscopy, and the TCID_50_ was calculated according to the method of Spearman–Kärber (100).

### Immunofluorescence labeling

Cytospin slides from the cell suspension aliquots were prepared with the Cytospin 4 Cytocentrifuge (Thermo Fisher Scientific) according to the manual. Subsequently, slides were fixed with 4% (wt/vol) paraformaldehyde for 20 minutes and kept at −80°C until use. Cytospin slides of uninfected and persistently CDV Ond-infected DH82 cells were used as negative and positive controls, respectively. Frozen slides were thawed slightly and washed in PBS containing 0.25% (vol/vol) Triton X-100 (Sigma-Aldrich). Afterward, unspecific binding of the respective secondary antibody was blocked by incubation in 20% (vol/vol) normal goat serum in PBS with 3% (wt/vol) bovine serum albumin (BSA) and 0.25% (vol/vol) Triton X-100 for 15 minutes. Primary antibodies anti-Iba1 (FUJIFILM) and anti-CDV-N (Table S4) were concurrently diluted in PBS with 3% (wt/vol) BSA and 0.25% (vol/vol) Triton followed by an overnight incubation at 4°C. Negative controls were incubated with normal rabbit serum and ascites fluid from non-immunized BALB/c mice instead of the primary antibodies. Slides were washed in PBS with 0.25% (vol/vol) Triton X-100 and incubated in the dark for 2 hours at room temperature with a secondary polyclonal antibody at a dilution factor of 1:200. Alexa Fluor 488-conjugated goat anti-rabbit (Jackson ImmunoResearch Europe) and Cy3-conjugated goat anti-mouse (Jackson ImmunoResearch Europe) were used to visualize signals. For staining of CC3, a primary labeled antibody was used (Table S4). After thawing, slides were rinsed with PBS thrice, and unspecific binding of the secondary antibody was blocked by incubation in 20% (vol/vol) normal goat serum in PBS with 3% (wt/vol) BSA and 0.25% (vol/vol) Triton X-100 for 60 minutes. Subsequently, the diluted antibody was applied and incubated overnight. Lastly, slides were washed in PBS and mounted with fluorescence mounting medium containing DAPI (Dako), followed by storage at 4°C in the dark. Representative fluorescence pictures for the figures were taken using a Keyence BZ-X800 microscope (Keyence).

### Immunohistochemistry and immunofluorescence for the detection of CDV antigen in lung tissues derived from naturally infected dogs

In order to confirm natural CDV infection of AMs, immunohistochemistry and immunofluorescence of lung tissue from five naturally infected dogs was performed. Lung tissue was taken at necropsy and fixed in 4% buffered formaldehyde solution, processed routinely, and embedded in paraffin. Sections were cut to a thickness of 2 µm, deparaffinized using Roticlear (Carl Roth), and rehydrated through a graded alcohol series. Endogenous peroxidase activity was suppressed by treating the samples with 0.5% hydrogen peroxide in 85% ethanol. Pretreatment involved a 20-minute incubation in citrate buffer (pH 6.0) using a microwave at 800 W, followed by blocking nonspecific bindings with goat normal serum (1:5) for 30 minutes. The primary antibody, mouse anti-CDV-N (Table S4), was applied overnight at 4°C, while negative control samples were treated with ascites fluid from nonimmunized BALB/c mice. The secondary antibody, goat anti-mouse (Vector Laboratories), was applied at a 1:200 dilution in PBS and incubated for 45 minutes at room temperature. This was followed by incubation with the avidin-biotin complex (Vectastain Elite ABC kit, Vector Laboratories) for 20 minutes at room temperature. Antigen-antibody interactions were visualized using 3,3′-diaminobenzidine tetrahydrochloride with 0.03% hydrogen peroxide for 5 minutes, and the slides were counterstained with Mayer’s hemalum for 30 seconds.

For immunofluorescence double labeling of lung tissue slides, deparaffinization, rehydration, and pretreatment were performed as mentioned above. To minimize non-specific binding, the slides were incubated for 30 minutes with 20% goat normal serum in PBS containing 1% BSA and 0.1% Triton X-100 (Sigma-Aldrich). Primary antibodies against Iba1 (Invitrogen) and CDV-N (Table S4) were simultaneously diluted in PBS with 1% BSA and 0.1% Triton X-100 and incubated overnight at 4°C, while negative controls were treated as mentioned above. Signal visualization was achieved using secondary polyclonal antibodies (Alexa Fluor 488-conjugated goat anti-mouse and Cy3-conjugated goat anti-rabbit; Jackson ImmunoResearch Europe) diluted 1:200 in PBS with 1% BSA and 0.1% Triton X-100, followed by a 45-minute incubation at room temperature in the dark. After washing with distilled water, autofluorescence was reduced using the Vector TrueVIEW Autofluorescence Quenching Kit (Vector Laboratories). Nuclei were visualized with Bisbenzimide Hoechst 33,258 (1:100 in sterile double-distilled water; Sigma-Aldrich Chemie), and the slides were mounted using fluorescence mounting medium (Dako).

### Digital image analysis

For quantification of Iba1^+^ cells, infection rates, and expression of MX1, ISG15, and CC3 on Cytospin slides, digitization was performed using an Olympus VS200 Digital slide Scanner (Olympus Europe). Image analysis was performed with the open-source software QuPath (version 0.4.3) (101). Total cell count was determined by using the “cell detection” tool in the DAPI channel. Afterward, object classifiers for both Iba1^+^ (macrophages), CDV^+^, MX1^+^, ISG15^+^, and CC3^+^ cells were trained to determine the number of single or double-positive cells. To quantify formation for syncytial cells, Iba1^+^ cells with three or more nuclei on digitized double-labeled Cytospin slides were counted manually using the “counting” tool in QuPath.

### RNA isolation and reverse transcription

Total RNA was isolated using the RNeasy Micro Kit (Qiagen) according to the manufacturer’s instructions including an on-column DNA digestion. RNA quality and concentration were measured with a Multiskan GO microplate spectrophotometer (µDrop plate, SkanIt software version 5.0.0.42, Thermo Fisher Scientific), and RNA was stored at −80°C until further use. For transcription of total RNA in complementary DNA (cDNA), the Sensiscript RT Kit (Qiagen) supplemented with RNaseOUT Recombinant Ribonuclease Inhibitor (Thermo Fisher Scientific) and random primers (Promega Corporation) was used following the supplier’s protocol.

### Generation of standard dilutions

To amplify gene products for the generation of standard dilutions, primer sequences for glyceraldehyde-3-phosphate dehydrogenase (GAPDH), TNF-α, and IL-6 were obtained from previous studies (93, 102), (Table S5). Plasmids based on the pEX-A128 vector containing under 300 bp of the respective canine cDNA genome sequences of IL-1β, IL-8, IL-10, IL-12, TGF-β, and IFN-γ (purchased from Eurofins Genomics) were used to generate standard dilutions (Table S6). In addition, cDNA which had been isolated from naturally CDV-infected canine lung tissue (GAPDH) or lymph nodes from uninfected dogs (TNF-α, IL-6) was also used to produce standard dilutions via PCR. The mastermix for PCR amplification contained Taq DNA Polymerase (Invitrogen, Thermo Fisher Scientific) with 1.5 mmol/L MgCl2, 0.2 mmol/L dNTP mix (New England Biolabs), and 300 nmol/L of each primer. A T-Gradient thermocycler (Biometra) was used with 40 cycles of an initial denaturing step of 94°C, an annealing step of 58°C (TNF-α, IL-6) or 59°C (GAPDH) for 45 seconds and elongation at 72°C for 40 seconds. Visualization of PCR was achieved by agarose gel electrophoresis. The respective band was extracted with NucleoSpin Gel and PCR Clean-up Kit (Macherey-Nagel) according to the manufacturer’s instructions. Absorbance at 260 nm was measured with a Multiskan GO microplate spectrophotometer to calculate the DNA concentration. PCR products or plasmids were diluted in DNase and RNase-free water to concentrations ranging from 10^2^ to 10^8^ copies/µL.

### Reverse transcription quantitative PCR

For RT-qPCR detection of canine GAPDH, TNF-α, IL-6, IL-10, IL-12, TGF-β, and IFN-γ, previously published primer sequences were used (48, 103–105) (Table S7). The Primer-BLAST software tool by the National Library of Medicine (106) was used to design analogous primers for IL-1β and IL-8 (Eurofins Genomics) (Table S7). RT-qPCR assays were performed using the AriaMx Real-Time PCR System (Agilent Technologies; Agilent Aria software version 1.71). The standard dilution series was included in every experimental setup to determine the copy numbers using the Brilliant III Ultra-Fast SYBR Green QPCR Master Mix (Agilent Technologies) according to the manufacturer’s instructions. Primers were added at a concentration of 200 nmol/L and carboxy-X-rhodamine served as a reference dye. Annealing steps were performed at 56°C (IL-1β), 60 °C (TNF-α), or 64 °C (GAPDH). Calculated copy numbers were normalized with the housekeeping gene GAPDH.

### Total RNA-seq

Quality and integrity of total RNA was assessed using an Agilent Technologies 2100 Bioanalyzer (Agilent Technologies). The RNA-seq library was generated from 50 ng total RNA using NEBNext Single Cell/Low Input RNA Library Prep Kit for Illumina (New England BioLabs) according to manufacturer’s protocols. The libraries were sequenced on an Illumina NovaSeq 6000 using NovaSeq 6000 S1 Reagent Kit (100 cycles, paired-end run) with an average of 3 × 10^7^ reads per RNA sample. A quality report was generated by FASTQC tool. Each sequence in the raw FASTQ files was trimmed on base call quality and sequencing adapter contamination using fastq-mcf (http://expressionanalysis.github.io/ea-utils/). Reads shorter than 15 bp were removed from FASTQ files. Trimmed reads were aligned to reference genomes (dog: ROS_Cfam_1.0, ensemble database version 105; CDV strain R252: Genbank Acc. KF640687; CDV strain Ond: Genbank Acc. AF378705) using open source short read aligner STAR (107). The subsequent analysis was performed using R version 4.2.1 (23 June 2022; https://www.R-project.org/). Raw count table was annotated using the biomaRt package (version 2.54.1) (108). For filtering, features with annotation type “rRNA” or “pseudogene” were removed from the data set and libraries in which the size differed by more than three standard deviations. For normalization of library sizes, the calcNormFactors function was used to find a set of scaling factors for the library sizes that minimizes the log-fold changes between the samples for most genes (109). Differential expression analysis for multifactor experiments was performed utilizing the generalized linear models (glm)-based statistical method of the edgeR Bioconductor package (version 3.38.4) (110). Dispersion was estimated using the Cox-Reid profile-adjusted likelihood method. Trended dispersions were estimated prior to estimating tagwise dispersions. After dispersion estimation, negative binomial glm were fitted to the data, after which the DEGs were determined using quasi-likelihood F-test. The statistical analysis to identify differential gene expression was performed using a multivariate regression model. In the following, a heatmap plot (threshold: absolute FC > 1, false discovery rate [FDR] < 0.05) was generated using the ComplexHeatmap package (version 2.12.1) (111), and GO enrichment analysis (“biological process”) and KEGG pathway analysis were performed from DEGs with the ClusterProfiler function (version 4.6.2) (112). Heatmaps of smaller subsets of genes were generated in R studio (version 2023.12.1) with the function heatmap2 of package gplots (version 3.1.3; https://github.com/talgalili/gplots). Venn’s diagrams were created using R package VennDiagram (version 1.7.3) (113).

### LDH assay

To determine cell viability, LDH activity in the supernatants was determined in triplicate using the Cytotoxicity Detection Kit (Roche) according to the manufacturer’s instructions. The absorbance at 492 nm and 630 nm (reference) was measured three times using a microplate reader (Fluostar Optima, BMG Labtech). Arithmetic means were calculated for each supernatant and the mean of the medium-only control as well as the absorbance value of the reference measurement were subtracted from each value.

### Quantitative sandwich-ELISA

Sandwich-ELISAs were used to quantify TNF-α and IFN-α secretions in supernatants of AM cultures, following the manufacturer’s instructions. The Quantikine ELISA Canine TNF-α Immunoassay (Bio-Techne) and the canine IFN alpha ELISA Kit (Invitrogen) were used. All standards, samples, and controls were measured in duplicates. For antigen binding, samples, standards, and controls were pipetted in 96-well plates coated with specific antibodies to canine TNF-α and IFN-α, respectively, followed by an incubation period. For measurement of TNF-α, samples were diluted 1:3. Bound antigen was labeled using biotinylated detection antibodies and streptavidin-horseradish peroxidase. Colorimetric quantification was achieved by adding a chromogen and measuring absorbance at 450 nm and 540 nm (background) with the SpectraMax ABS Plus reader (Molecular Devices). Using Softmax Pro software (version 7.2, Molecular Devices), four-parameter logistic standard curves were fitted to the standards, and absorbance values were plotted to determine TNF-α and IFN-α concentration, respectively, after subtraction of background absorbance. Mean values were calculated of all duplicates, and TNF-α values were multiplied with the dilution factor.

### Apoptosis/necrosis assay

To investigate cell death in CDV-infected AMs, differential staining of cells to distinguish between live, apoptotic, and necrotic cells was performed, using the Apoptosis/Necrosis Assay Kit (Abcam) according to the manufacturer’s instructions with slight modifications. Briefly, 50,000 cells per well were grown in a black 96-well plate with clear bottom, and at 1 dpi, staining was performed. Two washing steps with RPMI 1640 medium without phenol red (Thermo Fisher Scientific) including centrifugation of the plate were performed and subsequently, a staining mix containing 2 µL/well Apopxin Green Indicator, 1 µL/well 7-AAD 200×, and 1 µL/well CytoCalcein Violet 450 was added. After a 45-minute incubation period in the dark, the plate was washed three times. After a final centrifugation step, five images per well were taken with the Keyence BZ-X800 microscope at 10× magnification, and the number of live, apoptotic, and necrotic cells was determined using the manual counting tool in QuPath version 0.5.1.

### Statistical analysis and graph design

R statistic program was used to perform statistical analysis, and box plots were generated with GraphPad Prism 10 (GraphPad Software). The presence of significant differences was tested using the non-parametric Kruskal–Wallis test. Subsequently, pairwise comparisons among groups were performed with multiple two-tailed Mann-Whitney *U* tests with the FDR adjustment of Benjamini and Hochberg for multiple group comparisons. Significance was assumed at a *q* value of 0.05 or smaller.

## Data Availability

The total RNA-seq data generated in this study can be accessed in GEO under accession no. GSE283373 (https://www.ncbi.nlm.nih.gov/geo/query/acc.cgi?acc=GSE283373).

## SUPPLEMENTAL MATERIAL

Figure S1 jvi.01761-25-s0001.tif Immunohistochemical and immunofluorescence staining of lung tissue from a dog naturally infected with CDV.

Figure S2 jvi.01761-25-s0002.tif Cytokine mRNA expression in CDV-infected canine AMs measured by RT-qPCR assays.

Figure S3 jvi.01761-25-s0003.tif Normalized CDV gene expression.

Figure S4 jvi.01761-25-s0004.tif Downregulation of genes related to cell homeostasis and interaction with other immune cells in cluster 2.

Supplemental material jvi.01761-25-s0005.docx Legends for Fig. S1 to S4; Tables S4 to S7.

Table S1 jvi.01761-25-s0006.xlsx Differentially expressed genes in clusters 1 to 6.

Table S2 jvi.01761-25-s0007.xlsx Enrichment analysis using GO terms annotated to biological function.

Table S3 jvi.01761-25-s0008.xlsx Enrichment analysis using KEGG pathways.

ASM does not own the copyrights to Supplemental Material that may be linked to, or accessed through, an article. The authors have granted ASM a non-exclusive, world-wide license to publish the Supplemental Material files. Please contact the corresponding author directly for reuse.
