# Supplementary figures and images for: Attenuation of canine distemper virus leads to a potent antiviral innate immune response with restricted infection of alveolar macrophages
Source: J Virol. 2025 Dec 17;100(1):e01761-25. doi: 10.1128/jvi.01761-25 (PMC12817944; doi:10.1128/jvi.01761-25)

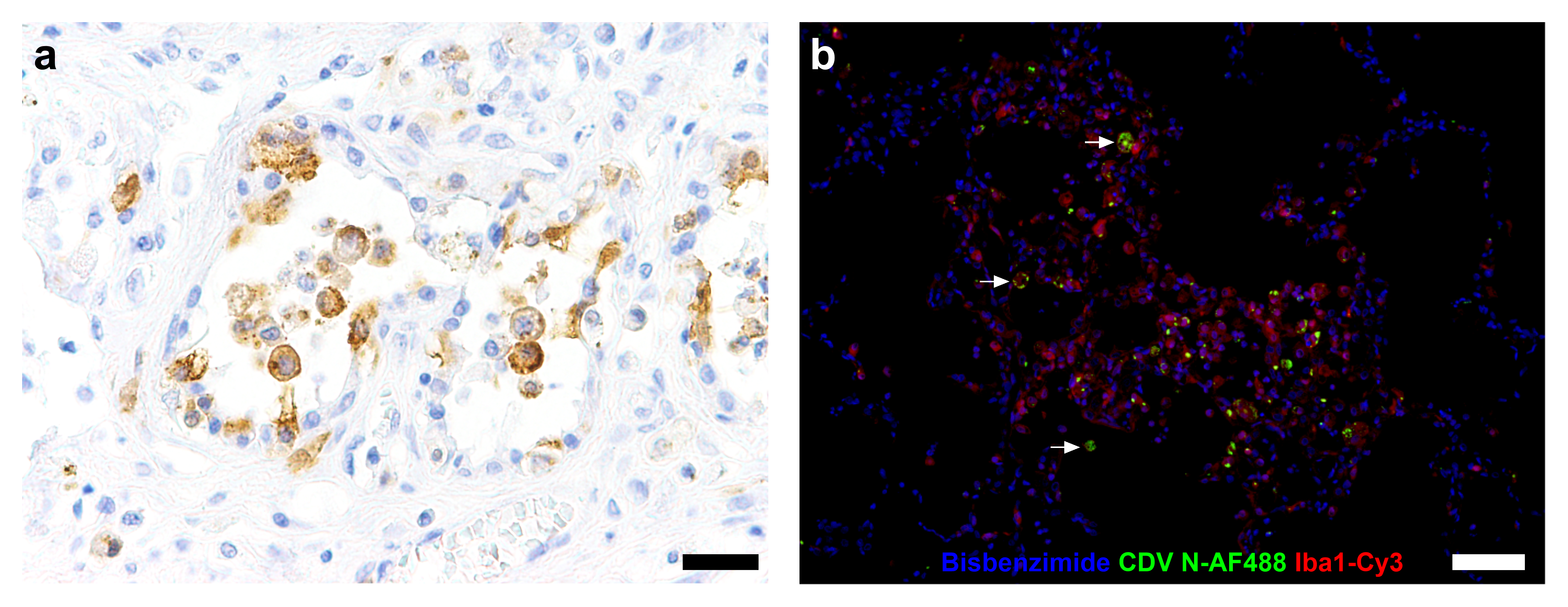

Supplement: Figure S1 — Immunohistochemical and immunofluorescence staining of lung tissue from a dog naturally infected with CDV. [file jvi.01761-25-s0001.tif]

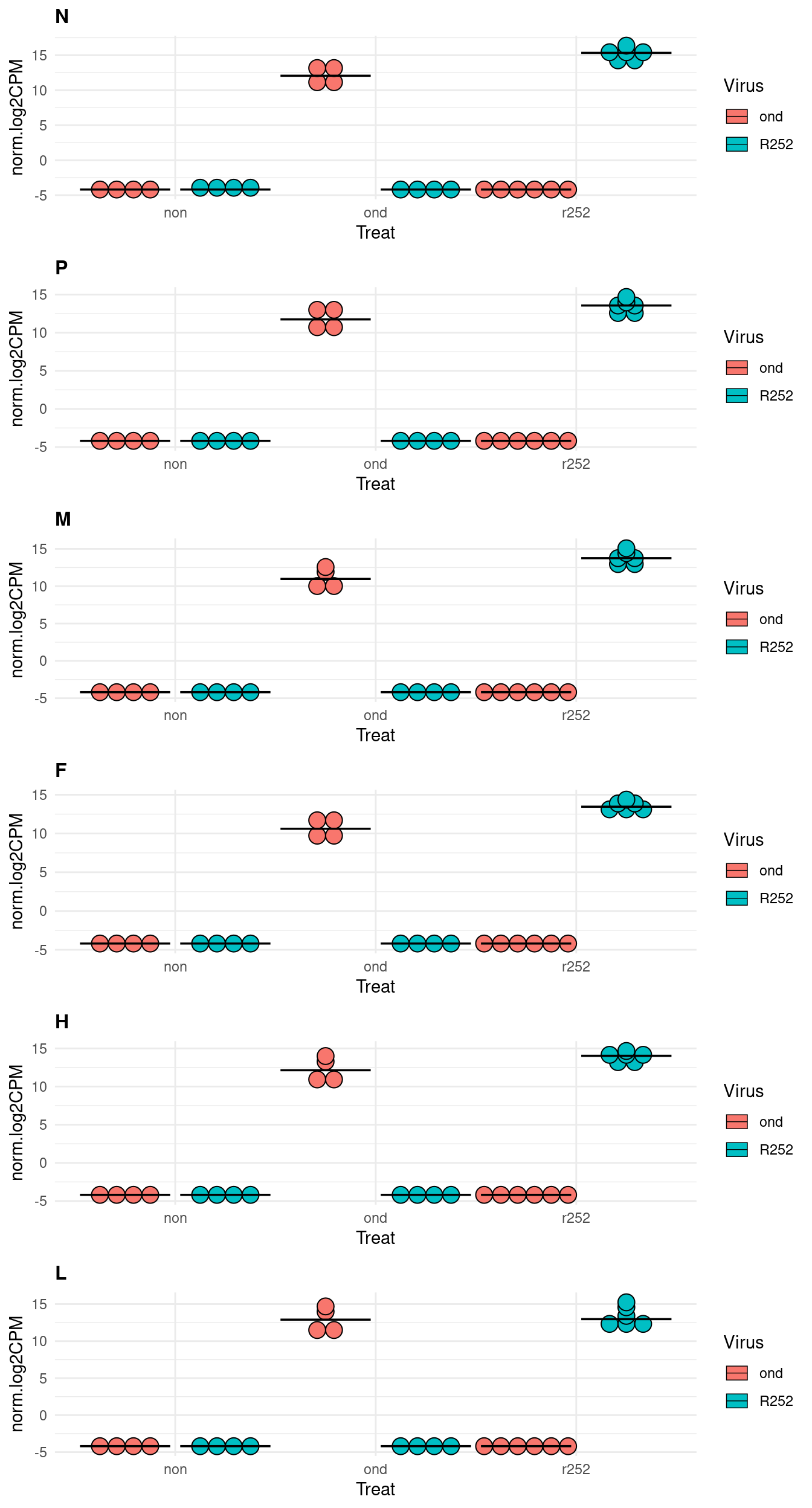

Supplement: Figure S3 — Normalized CDV gene expression. [file jvi.01761-25-s0003.tif]
