# Supplementary material for: Attenuation of canine distemper virus leads to a potent antiviral innate immune response with restricted infection of alveolar macrophages
Source: J Virol. 2025 Dec 17;100(1):e01761-25. doi: 10.1128/jvi.01761-25 (PMC12817944; doi:10.1128/jvi.01761-25)
Supplement: Supplemental material — Legends for Fig. S1 to S4; Tables S4 to S7. [file jvi.01761-25-s0005.docx]

# **Supplementary Materials**

**Figure S1.** Immunohistochemical (**a**) and immunofluorescence (**b**) staining of lung tissue from a dog naturally infected with canine distemper virus (CDV) **a.** CDV nucleoprotein (N) within alveolar cells. **b.** Detection of CDV-N (green) within Iba1^+^ (red) alveolar macrophages (white arrows). Nuclear counterstaining by bisbenzimide Hoechst 33258 (blue). Scale bars = 20 µm (**a**), 100 µm (**b**).

**Figure S2.** Cytokine mRNA expression in canine distemper virus (CDV)-infected canine alveolar macrophages (AMs) measured by RT-qPCR assays. **a.** Interleukin (IL)-6. **b.** IL-8. **c.** IL-10. **d.** IL-12. **e.** Transforming growth factor (TGF)-β. **f.** Interferon (IFN)-γ. Data are shown as median with 95% confidence interval. Statistical analyses (Mann-Whitney *U* test) reveal no significant (p ≤ 0.05) differences among groups.

**Figure S3.** Normalized canine distemper virus (CDV) gene expression at 1 day post infection. Expression of CDV-Ond (red; Genbank Acc. AF378705) and CDV-R252 (green; Genbank Acc. KF640687) genes in infected alveolar macrophages, normalized to host transcripts, by mapping of reads to the boundaries of the N (nucleoprotein; 108-1679 bp), P (phosphoprotein; 1801-3324 bp), M (matrix protein; 3432-4439 bp), F (fusion protein; 4935-6923 bp), H (haemagglutinin; 7079-8902 bp) and L (polymerase protein; 9030-15584 bp) genes. Data reveal no significant expression differences between both CDV strains.

**Figure S4.** **a-b.** Downregulation of genes related to cell homeostasis and interaction with other immune cells in *cluster 2*. **a.** Heatmap showing expression of DEGs in *cluster 2* in GO:0002703 *“regulation of leukocyte mediated immunity”*. **b.** Heatmap showing expression of DEGs in *cluster 2* in GO:0030036 *“actin cytoskeleton organization”*. **c-d.** Differential expression of genes associated with cellular metabolism in canine distemper virus (CDV)-infected AMs. **c.** Heatmap showing expression of DEGs in *cluster 1* in KEGG cfa 01212 *“fatty acid metabolism”*. **d.** Heatmap showing expression of DEGs in *cluster 1* in GO:0009260 *“ribonucleotide biosynthetic process”*.

**TableS1-3:** gene lists, see extra excel-files.

**Table S4.** Primary antibodies used for immunohistochemistry and immunofluorescence

| **Epitope** | **Specificity** | **Source** | **Cat No** | **Species** | **Clone** | **Dilution** |
| --- | --- | --- | --- | --- | --- | --- |
| CDV-N | CDV-infected cells | A. Zurbriggen, University of Bern, Bern, Switzerland | - | mouse | D110 | 1:1000 (IHC)  1:100 (IF) |
| Iba1 | histiocytic cells | Invitrogen™, Thermo Fisher Scientific, Langenselbold, Germany | PA5-27436 | rabbit | polyclonal | 1:500 |
| Iba1 | histiocytic cells | FUJIFILM Wako Pure Chemical Corporation, Osaka, Japan | 019-19741 | rabbit | polyclonal | 1:250 |
| MX1 | MX1 protein | Prof. Dr. Haller and PD Dr. Kochs, University Medical Center Freiburg, Freiburg, Germany | 11/2005/S417 | mouse | MX143 | 1:500 |
| ISG15 | ISG15 protein | Santa Cruz Biotechnology, Santa Cruz, CA, USA | Sc-166755 | mouse | F-9 | 1:200 |
| CC3 | cleaved caspase-3 | Cell Signaling Technology, Cambridge, United Kingdom | 9602S | rabbit | Asp174 D3E9 | 1:100 |

**Table S5**. Primers for RT-PCR for generation of standard dilutions

| **Gene** | **Primer direction** | **Primer sequence (5’-3’)** | **Position** | **Amplicon length (bp)** | **Genebank accession number** | **Reference** |
| --- | --- | --- | --- | --- | --- | --- |
| GAPDH | forward | AAG GTC GGA GTC AAC GGA TT | 7-26 | 365 | AB038240 | (1) |
|  | reverse | GCA GAA GGA GCA GAG ATG ATG | 371-351 |  |  |  |
| IL-6 | forward | TCT CCA CAA GCG CCT TCT CC | 68-87 | 318 | U12234 | (2) |
|  | reverse | TTC TTG TCA AGC AGG TCT CC | 385-366 |  |  |  |
| TNF-α | forward | CCA AGT GAC AAG CCA GTA GC | 32-51 | 274 | Z70046 | (2) |
|  | reverse | TCT TGA TGG CAG AGA GTA GG | 305-287 |  |  |  |

**Table S6.** Plasmids used for generation of standard dilutions

| **Gene** | **Plasmid sequence (5’-3’)** | **Position** | **Gene size (bp)** | **Genebank accession number** | **Reference** |
| --- | --- | --- | --- | --- | --- |
| IL-1β | TGC AAG TTA CAG GAC ATA AGC CAC AAA TAC CTG GTG CTG TCT AAC TCA TAT GAG CTT CGG GCT CTC CAC CTC AAT GGG GAA AAT GTG AAC AAA CAA GTG GTG TTC CAC ATG AGC TTT GTG CAC GGG GAT GAA AGT AAT AAC AAG ATA CCT GTG GTC TTG GGC ATC AAA CAA AAG AAT CTG TAC CTG TCC TGT GTG ATG AAG GAT GGA AAG CCC ACC CTA CAG CTA GAG AAG GTA GAC CCC | 361-600 | 240 | NM_001037971.1 |  |
| IL-8 | AGT TCA GAA CTT CGA TGC CAG TGT ATA AAA ACA CAC TCC ACA CCT TTC CAT CCC AAA TAT ATT AAA GAA CTG AGA GTG ATT GAC AGT GGC CCA CAT TGT GAA AAC TCA GAA ATC ATT GTA AAG CTT TTC AAT GGA AAT GAG GTG TGC CTG GAC CCC AAG GAA AAA TGG GTA CAA AAG GTT GTG CAG ATA T | 91-280 | 190 | U10308.1 |  |
| IL-10 | ATG CCC CGG GCT GAG AAC CAC GAC CCA GAC ATC AAG AAC CAC GTG AAC TCC CTG GGA GAG AAG CTC AAG ACC CTC AGG CTG AGA CTG AGG CTG CGA CGC TGT CAC CGA TTT CTT CCC TGT GAG AAT AAG AGC AAG GCG GTG GAG CAG GTG AAG AGC GCA TTT AG | 287-450 | 164 | U33843 | (3) |
| IL-12 | ATG CAT CCT CAG CAG TTG GT C ATC TCC TGG TTT TCC CTC GTT TTG CTG GCG TCT TCC CTC ATG ACC ATA TGG GAA CTG GAG AAA GAT GTT TAT GTT GTA GAG TTG GAC TGG CAC CCT GAT GCC CCC GGA GAA ATG GTG GTC CTC ACC TGC CAT ACC CCT GAA GAA GAT GAC ATC ACT TGG ACC TCA GCG CAG AGC AGT GAA GTC CTA GGT TCT GGT AAA ACT CTG ACC ATC CAA GTC AAA GAA TTT GGA GAT GCT GGC CAG TAT ACC TGC CAT AAA GGA | 1-279 | 279 | U49100 | (3) |
| TGF-β | GGA GCT GTA CCA GAA ATA TAG CAA TGA TTC CTG GCG CTA CCT CAG CAA CCG GCT GCT GGC GCC CAG CGA CAC GCC AGA ATG GCT GTC CTT TGA TGT CAC TGG AGT CGT GAG GCA GTG GCT GAG CCA TGG AGG GGA AGT CGA GGG CTT TCG CCT CAG TGC CCA CTG TTC CTG TGA CAG CAA AGA TAA CAC A | 561-750 | 190 | L34956 | (3) |
| IFN-γ | CCA GAT GTA TCG GAC GGT GGG TCT CTT TTC GTA GAT ATT TTG AAG AAA TGG AGA GAG GAG AGT GAC AAA ACA ATC ATT CAG AGC CAA ATT GTC TCT TTC TAC TTG AAA CTG TTT GAC AAC TTT AAA GAT AAC CAG ATC ATT CAA AGG AGC ATG GAT ACC ATC AAG GAA GAC ATG CTT GGC AAG TTC TTA AAT AGC AGC ACC AGT AAG AGG GAG GAC TTC CTT AAG CTG ATT CAA ATT CCT GTG AAC GAT CTG CAG GTC CAG CGC AAG GCG ATA A | 76-349 | 274 | S41201 | (3) |

**Table S7.** Primers for RT-qPCR assays

| **Gene** | **Primer direction** | **Primer sequence (5’-3’)** | **Position** | **Amplicon length (bp)** | **Genebank accession number** | **Reference** |
| --- | --- | --- | --- | --- | --- | --- |
| GAPDH | forward | GTC ATC AAC GGG AAG TCC ATC TC | 196-218 | 84 | AB038240 | (4) |
|  | reverse | AAC ATA CTC AGC ACC AGC ATC AC | 279-257 |  |  |  |
| IL-1β | forward | TCG GGC TCT CCA CCT CAA TG | 517-536 | 158 | NM_001037971.1 | Primer Blast (5) |
|  | reverse | TGG GCT TTC CAT CCT TCA TCA C | 674-653 |  |  |  |
| IL-6 | forward | TGA TGC CAC TTC AAA TAG TCT ACC A | 156-180 | 89 | U12234 | (6) |
|  | reverse | TCA GTG CAG AGA TTT TGC CGA GGA | 244-221 |  |  |  |
| IL-8 | forward | ACA CTC CAC ACC TTT CCA TCC C | 123-144 | 122 | U10308.1 | Primer Blast (5) |
|  | reverse | GGT CCA GGC ACA CCT CAT TTC C | 244-223 |  |  |  |
| IL-10 | forward | ACC ACG ACC CAG ACA TCA AGA A | 303-324 | 120 | U33843 | (3) |
|  | reverse | CCT TGC TCT TAT TCT CAC AGG GAA G | 422-398 |  |  |  |
| IL-12 | forward | CTC GTT TTG CTG GCG TCT TC | 37-56 | 153 | U49100 | (3) |
|  | reverse | CGC TGA GGT CCA AGT GAT GT | 189-170 |  |  |  |
| TNF-α | forward | GGA GCT GAC AGA CAA CCA GCT GA | 133-155 | 91 | Z70046 | (6) |
|  | reverse | GGA AGG GCA CCC TTG GCC CT | 223-204 |  |  |  |
| TGF-β | forward | TGG CGC TAC CTC AGC AAC CG | 592-611 | 115 | L34956 | (6) |
|  | reverse | AGC CCT CGA CTT CCC CTC CA | 706-687 |  |  |  |
| IFN-γ | forward | AGC ATG GAT ACC ATC AAG GAA GA | 223-245 | 104 | S41201 | (7) |
|  | reverse | AGA TCG TTC ACA GGA ATT TGA ATC A | 326-302 |  |  |  |

# **References (Supplementary Materials)**

1. Puff C, Krudewig C, Imbschweiler I, Baumgärtner W, Alldinger S. 2009. Influence of persistent canine distemper virus infection on expression of RECK, matrix-metalloproteinases and their inhibitors in a canine macrophage/monocytic tumour cell line (DH82). Vet J 182:100-7.

2. Gröne A, Frisk AL, Baumgärtner W. 1998. Cytokine mRNA expression in whole blood samples from dogs with natural canine distemper virus infection. Vet Immunol Immunopathol 65:11-27.

3. Chludzinski E, Klemens J, Ciurkiewicz M, Geffers R, Pöpperl P, Stoff M, Shin DL, Herrler G, Beineke A. 2022. Phenotypic and transcriptional changes of pulmonary immune responses in dogs following canine distemper virus infection. Int J Mol Sci 23: 10019.

4. von Smolinski D, Leverkoehne I, von Samson-Himmelstjerna G, Gruber AD. 2005. Impact of formalin-fixation and paraffin-embedding on the ratio between mRNA copy numbers of differently expressed genes. Histochem Cell Biol 124:177-88.

5. Ye J, Coulouris G, Zaretskaya I, Cutcutache I, Rozen S, Madden TL. 2012. Primer-BLAST: a tool to design target-specific primers for polymerase chain reaction. BMC Bioinformatics 13:134.

6. Spitzbarth I, Bock P, Haist V, Stein VM, Tipold A, Wewetzer K, Baumgärtner W, Beineke A. 2011. Prominent microglial activation in the early proinflammatory immune response in naturally occurring canine spinal cord injury. J Neuropathol Exp Neurol 70:703-14.

7. Schwartz M, Puff C, Stein VM, Baumgärtner W, Tipold A. 2011. Pathogenetic factors for excessive IgA production: Th2-dominated immune response in canine steroid-responsive meningitis-arteritis. Vet J 187:260-6.
